# NMR-Based Metabolomic Analysis of Biotic Stress Responses in the Traditional Korean Landrace Red Pepper (*Capsicum annuum* var. *annuum*, cv. Subicho)

**DOI:** 10.3390/ijms25189903

**Published:** 2024-09-13

**Authors:** Gi-Un Seong, Dae-Yong Yun, Dong-Hyeok Shin, Jeong-Seok Cho, Seul-Ki Park, Jeong Hee Choi, Kee-Jai Park, Jeong-Ho Lim

**Affiliations:** 1 Food Safety and Distribution Research Group, Korea Food Research Institute, Seongnam-si 55365, Republic of Korea; s.giun@kfri.re.kr (G.-U.S.); ydy0401@kfri.re.kr (D.-Y.Y.); s.donghyeok@kfri.re.kr (D.-H.S.); jscho@kfri.re.kr (J.-S.C.); choijh@kfri.re.kr (J.H.C.); jake@kfri.re.kr (K.-J.P.); 2 Smart Food Manufacturing Project Group, Korea Food Research Institute, Seongnam-si 55365, Republic of Korea; skpark@kfri.re.kr

**Keywords:** Subicho, red pepper, *Capsicum annuum*, NMR, metabolomics, biotic stress, stress resistance

## Abstract

Korean landrace red peppers (*Capsicum annuum* var. Subicho), such as the traditional representative Subicho variety, are integral to Korean foods and are often consumed raw or used as a dried powder for cuisine. However, the known vulnerability of local varieties of landrace to biotic stresses can compromise their quality and yield. We employed nuclear magnetic resonance (NMR) spectroscopy coupled with a multivariate analysis to uncover and compare the metabolomic profiles of healthy and biotic-stressed Subicho peppers. We identified 42 metabolites, with significant differences between the groups. The biotic-stressed Subicho red peppers exhibited lower sucrose levels but heightened concentrations of amino acids, particularly branched-chain amino acids (valine, leucine, and isoleucine), suggesting a robust stress resistance mechanism. The biotic-stressed red peppers had increased levels of TCA cycle intermediates (acetic, citric, and succinic acids), nitrogen metabolism-related compounds (alanine, asparagine, and aspartic acid), aromatic amino acids (tyrosine, phenylalanine, and tryptophan), and γ-aminobutyric acid. These findings reveal the unique metabolic adaptations of the Subicho variety, underscoring its potential resilience to biotic stresses. This novel insight into the stress response of the traditional Subicho pepper can inform strategies for developing targeted breeding programs and enhancing the quality and economic returns in the pepper and food industries.

## 1. Introduction

Korean landrace red peppers (*Capsicum annuum* var. *annuum*, cv. Subicho), such as the traditional Subicho variety, are members of the Solanaceae family and are integral to Korean cuisine. Renowned for their bold red color, pungent flavor, and excellent nutritional profile, these peppers contain a rich array of vitamins, minerals, and antioxidants [1]. Their versatility as a condiment has led to their widespread cultivation across numerous tropical and subtropical regions [2]. Over time, selective breeding has significantly improved the drying quality, size, yield, and disease resistance of red peppers, with only the most competitive varieties being widely available [3]. Despite these advances, traditional landrace varieties such as Subicho, which have been maintained by farmers for generations, remain highly valued for their superior adaptation to local climates and their desirable taste and market price [4,5].

Red peppers, including the Subicho cultivar, have diverse culinary applications and are consumed fresh, dried, or ground. They are especially favored in spicy seasonings, kimchi, and red pepper paste [6,7]. However, during the hot and humid harvest season, red peppers face significant challenges from biotic stresses, such as infections by phytopathogenic fungi, bacteria, viruses, and weeds. The high moisture content of red peppers during this harvest period, which often reaches 70–80%, significantly increases the vulnerability of these peppers to biotic stress and results in considerable quality and yield losses [8]. Among these stresses, anthracnose is particularly detrimental, often disfiguring peppers post-harvest and diminishing their visual appeal and spicy flavor, which are critical for consumer satisfaction [9,10]. The economic repercussions of these biotic stress-induced quality losses not only impact local consumption but also limit their export potential, thereby constraining the growth of the pepper industry [11,12].

Addressing these issues is crucial for improving the overall quality and economic viability of red pepper production. Metabolomics, which employs advanced analytical tools such as nuclear magnetic resonance (NMR) and liquid and gas chromatography–mass spectrometry, offers a rapid and reliable method for characterizing plant responses to various conditions, including biotic stress [13,14,15,16]. NMR is particularly valuable in metabolomics studies due to its high reproducibility, quantitative capabilities, and ability to analyze complex mixtures without the need for sample separation [17,18]. Although previous research has extensively explored the growth, environmental adaptations, and genetic improvements of peppers [19,20,21], the metabolomic differences between healthy and biotic-stressed red peppers at the point of harvest are not yet fully understood.

In this study, we focused on the Subicho variety and compared healthy Subicho peppers with those naturally infected by biotic stresses. By analyzing these peppers at the critical harvest stage, we aimed to directly assess the metabolomic changes associated with biotic stress.

## 2. Results and Discussion

### 2.1. Identification of Metabolites in Red Pepper Extracts Using 1D and 2D NMR Spectroscopy

**Figure 1:**
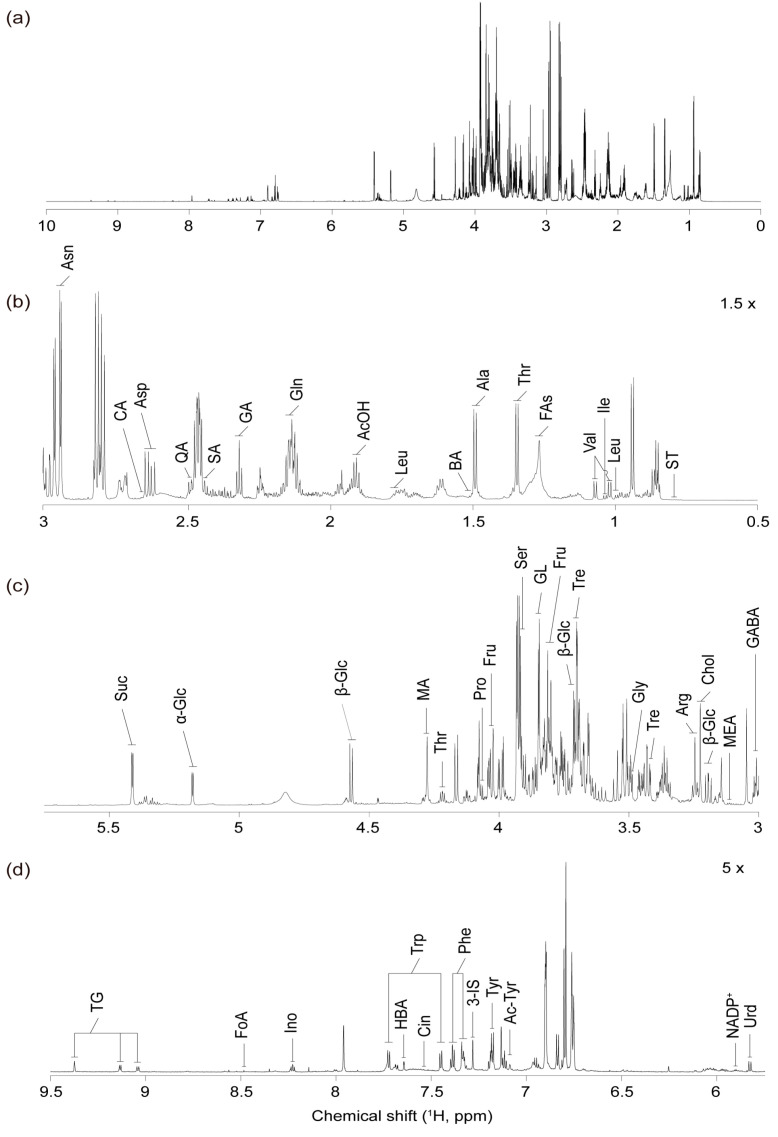
Characteristic ^1^H NMR spectrum (700 MHz) of the quality control sample prepared by pooling equal volumes of all the red pepper extracts: (**a**) the full spectrum (0–10 ppm), (**b**) the aliphatic or allylic region (0.5–3 ppm), (**c**) the alkene or heteroatom-attached region (3–5.6 ppm), and (**d**) the alkene or aromatic region (5.6–9.5 ppm). α-Glc—α-Glucose; β-Glc—β-Glucose; Suc—Sucrose; Fru—Fructose; Tre—Trehalose; Thr—Threonine; Ser—Serine; Asn—Asparagine; Gln—Glutamine; Ala—Alanine; Gly—Glycine; Pro—Proline; Val—Valine; Leu—Leucine; Ile—Isoleucine; Asp—Aspartic acid; GABA—γ-Aminobutyric acid; Arg—Arginine; Phe—Phenylalanine; Trp—Tryptophan; Tyr—Tyrosine; AcOH—Acetic acid; FoA—Formic acid; MA—Malic acid; SA—Succinic acid; GA—Glutaric acid; CA—Citric acid; QA—Quinic acid; HBA—Hydrobenzoic acid; Cin—Cinnamate; Chol—Choline; TG—Trigonelline; MEA—Ethanolamine; Ino—Inosine; 3-IS—3-Indoxylsulfate; Urd—Uridine; Ac-Tyr—Acetyl-tyrosine; NADP+—Nicotinamide adenine dinucleotide phosphate; ST—Sterols; GL—Glycerol; FAs—Fatty acids; and BA—Butyric acid.

The ^1^H NMR spectral analysis of the pericarp components from healthy Subicho and biotic-stressed Subicho red pepper samples revealed a diverse range of metabolites critical to the physiological and biochemical processes within the pepper tissues (Figure 1). The spectrum exhibited distinct regions, each corresponding to specific organic compounds involved in key metabolic pathways. A comprehensive metabolite profile (Figure 1) was established through meticulous investigation and validation using advanced 2D NMR techniques, including TOCSY and HSQC [22]. Additional details are provided in Table S1. The several amino acids that were identified in the chemical shift range of 0.5–3.0 ppm emphasized the central role of protein and nitrogen metabolism in the response of the red pepper to biotic stress. These amino acids included leucine, isoleucine, valine, threonine, alanine, glutamine, aspartic acid, and asparagine. Additionally, the prevalence of organic acids such as acetic, glutaric, succinic, quinic, and citric acids, along with sterols, fatty acids, and butyric acid reflects their involvement in various metabolic pathways and cellular functions. These organic acids play important roles in the tricarboxylic acid (TCA) cycle and stress-induced energy metabolism, which are critical for maintaining cellular homeostasis under biotic stress.

In the range of 3.0–5.7 ppm, signals corresponding to essential amino acids, including γ-aminobutyric acid, arginine, glycine, serine, and proline, were observed. This region also revealed signals from carbohydrates, including β-glucose, trehalose, fructose, α-glucose, and sucrose, underscoring the importance of carbohydrate metabolism in energy production and cellular processes in both healthy and biotic-stressed Subicho red peppers. Carbohydrates, particularly sucrose, are not only primary energy sources but also act as signaling molecules that modulate stress-related gene expression by linking metabolic and defense pathways. Furthermore, the presence of malic acid, ethanolamine, choline, and glycerol in this spectral region highlights their roles in biosynthetic pathways and cell membrane integrity. The detection of glycerol and choline can confirm enhanced phospholipid biosynthesis and membrane remodeling in response to stress, which is important for maintaining the cellular structure during pathogen invasion.

The relatively weaker signals detected in the 5.7–9.5 ppm range predominantly originate from the aromatic groups of amino acids and phenolic compounds. These signals were attributed to amino acids such as tyrosine, phenylalanine, and tryptophan, as well as organic acids such as cinnamate, hydroxybenzoic acid, and formic acid. The identification of phenolic compounds such as cinnamate and hydroxybenzoic acid is significant, as these metabolites are precursors to key plant defense compounds, including lignin and flavonoids, which reinforce plant cell walls and provide antimicrobial properties [23]. Additionally, this spectral region revealed the presence of compounds including uridine, nicotinamide adenine dinucleotide phosphate, acetyl-tyrosine, 3-indoxyl sulfate, inosine, and trigonelline. Despite their lower intensity, these signals provide valuable insights into the metabolic adaptations of nucleotides, and related compounds such as uridine and inosine can predict active nucleotide metabolism that may support enhanced RNA synthesis and repair mechanisms under stress conditions.

### 2.2. Multivariate Statistical Analysis of the ^1^H NMR Spectra

**Figure 2:**
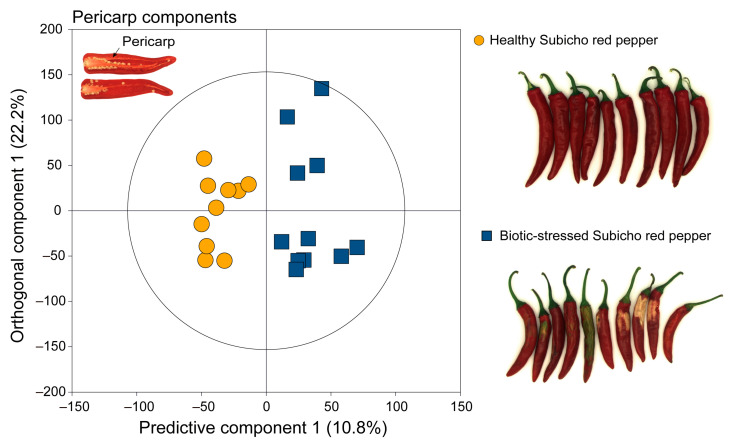
OPLS-DA score plots between healthy Subicho and biotic-stressed Subicho red peppers derived from the ^1^H NMR spectra (700 MHz).

**Figure 3:**
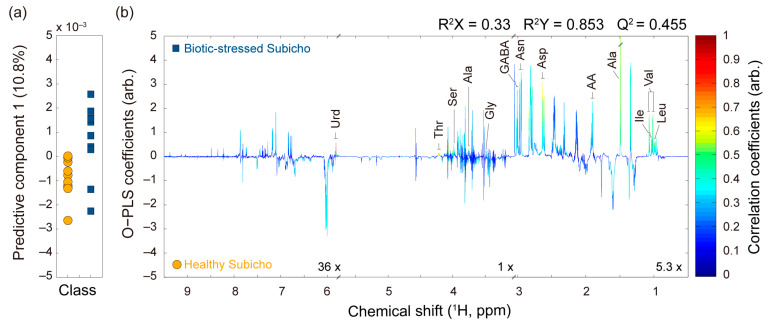
OPLS-DA score (**a**) and coefficients or loading plots (**b**) between the healthy Subicho and biotic-stressed Subicho red peppers to identify metabolites responsible for the metabolic differentiation. The color code in the loading plot corresponds to the correlation between the variables. All the OPLS-DA models were generated with one predictive and one orthogonal component. Their reliability and predictability are indicated by R^2^X, R^2^Y, and Q^2^. Abbreviations of the names of the assigned metabolites are listed in Table S1.

Chemometric tools were used to effectively manage the dataset’s complexity while providing classification capabilities that reduced noise and variability [24]. The OPLS-DA score plots presented a comprehensive visualization with a clear distinction between healthy Subicho and biotic-stressed Subicho red peppers (Figure 2). Although the PCA and PLS-DA results also revealed significant differences between the two groups, we found the OPLS-DA model to be the most effective for making the distinction, which is the reason why we presented only the OPLS-DA results in the main text. However, for completeness, the PCA and PLS-DA results have been included in the Supplementary Materials (Figure S1) for further reference. Moreover, each OPLS-DA model was subjected to statistical validation via a permutation analysis using 200 model permutations, which demonstrated a satisfactory predictive power without overfitting (Figure S2) [25]. The analysis revealed spectral differences between the healthy and biotic-stressed Subicho red peppers. Consequently, the OPLS-DA scores and loading plots were constructed to visually identify differences in the ^1^H NMR spectrum according to the biotic stress conditions in red peppers (Figure 3). The OPLS-DA loading plot results revealed that certain amino acids, such as threonine, serine, alanine, glycine, asparagine, valine, leucine, and isoleucine, were strongly correlated with the biotic-stressed Subicho red peppers. Correlations were also observed with their levels of uridine, γ-aminobutyric acid, and acetic acid. Thus, the occurrence and condition of biotic-stressed red peppers could be predicted by monitoring changes in these key amino acids and other metabolites. These findings contribute to our understanding of the biochemical processes associated with the biotic stress response in red peppers.

### 2.3. Metabolomics in Red Peppers Using ^1^H NMR Spectroscopy

**Figure 4:**
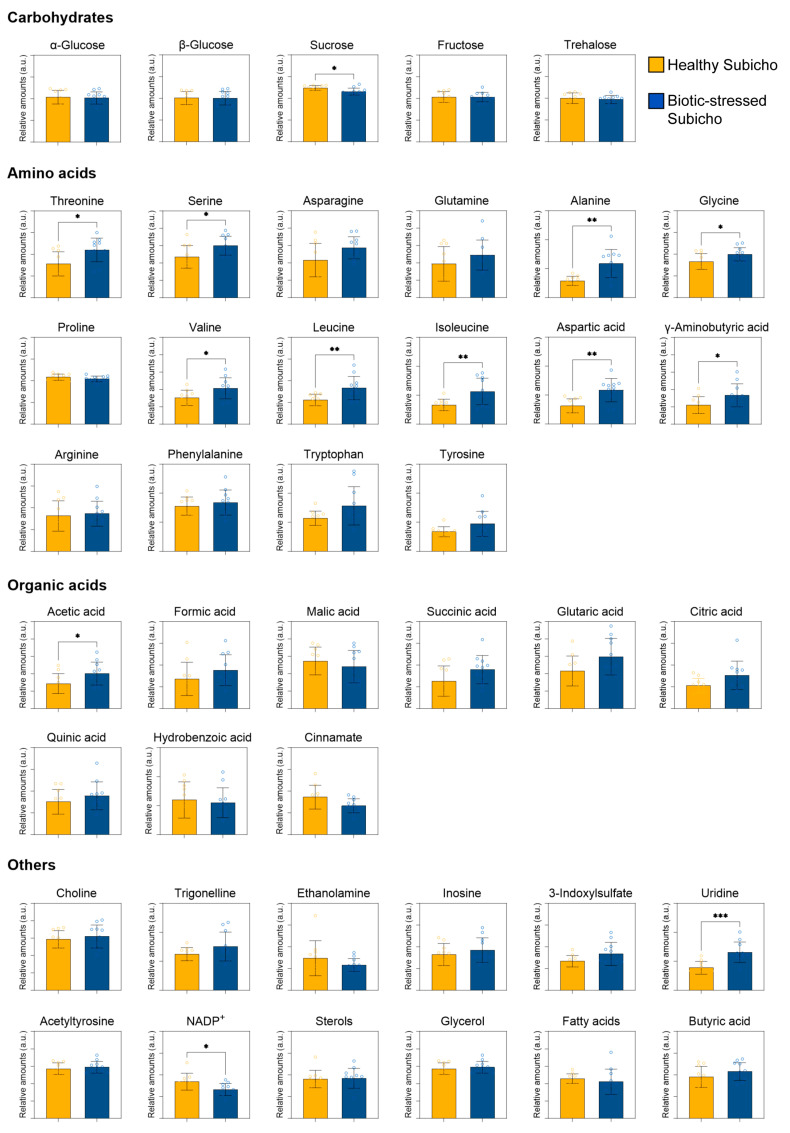
Relative quantification of the individual metabolites in red peppers, and a comparison of relative metabolite contents between the healthy Subicho and biotic-stressed Subicho red peppers. Asterisks represent statistical significance using the Student’s *t*-test (* *p* < 0.05, ** *p* < 0.01, *** *p* < 0.001). OPLS-DA score plots between healthy Subicho and biotic-stressed Subicho red peppers derived from the ^1^H NMR spectra (700 MHz).

**Figure 5:**
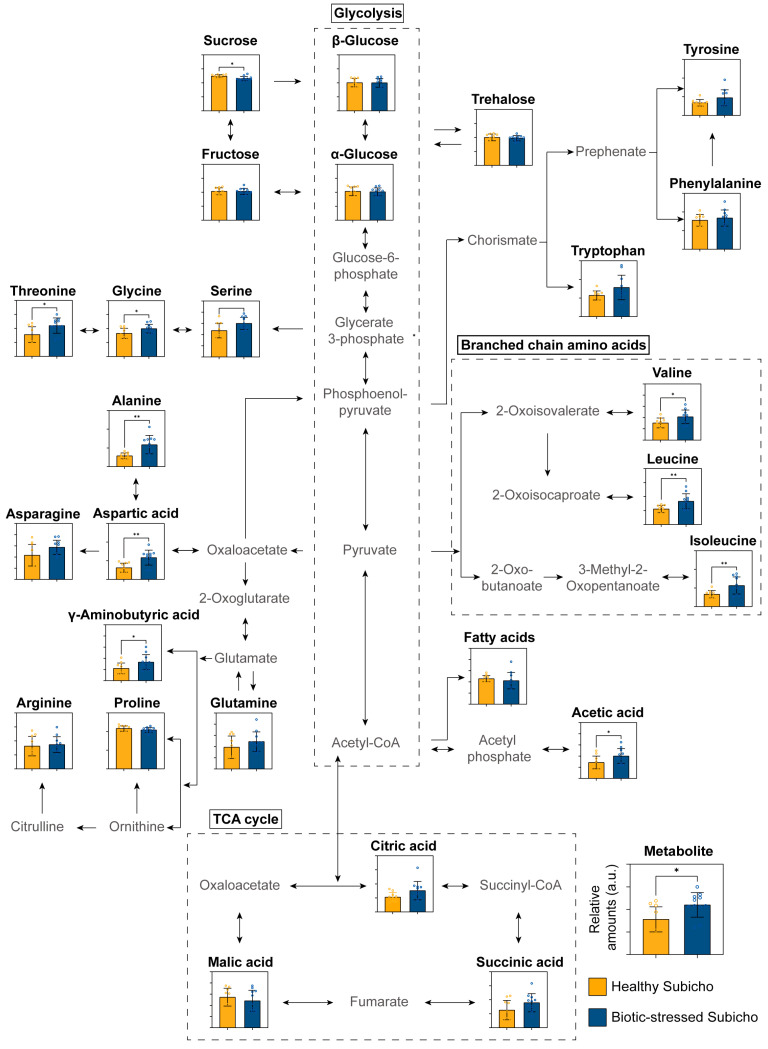
Schematic metabolic flux between the healthy Subicho and biotic-stressed Subicho red pepper. Asterisks represent statistical significance using the Student’s *t*-test (* *p* < 0.05, ** *p* < 0.01).

Figure 4 shows the relative amounts of various metabolites in the pericarps of the healthy Subicho and biotic-stressed Subicho red peppers. In addition, Figure 5 provides a detailed schematic of the metabolic changes based on the major distinctive relative amounts of the red pepper pericarp. Carbohydrates, which are essential for regulating the metabolism, play a critical role in maintaining the quality of the fruit, and serve as the primary energy sources throughout the pre- and post-harvest development and ripening stages. Typically, the levels of various mono-, di-, and small oligosaccharides, such as glucose, sucrose, and trehalose increase during plant development [26]. This increase is linked to enhanced plant resistance and defense mechanism activation, a phenomenon called “sweet immunity” or “sugar-enhanced defense” [27,28]. Studies have demonstrated that these sugars are not only energy sources but also signaling molecules that activate the genes related to plant defense. In this study, a significant discrepancy in carbohydrate levels, particularly for sucrose, in the pericarp components between the healthy and biotic-stressed Subicho red peppers was observed. This reduction in sucrose was further elucidated using insights from previous genetic and metabolic research on fruits, such as blueberries [29]. For example, blueberries that were infected with anthracnose exhibited genetic adaptations and metabolic alterations in response to the pathogen, which indirectly affected their sugar metabolism. These changes might have resulted in decreased sucrose levels in infected fruits as the plant reallocated resources to boost its defense mechanisms rather than store sugar. Furthermore, in bananas that were treated with melatonin to delay the onset of anthracnose, a comparative transcriptomic analysis revealed altered gene expression patterns that significantly impacted sugar metabolism pathways, further supporting the hypothesis that stress from disease can reduce sucrose accumulation [30]. In biotic-stressed Subicho red peppers, the decreased sucrose levels likely reflect a similar shift in metabolic priorities, where the energy and resources are redirected from storage to the activation of stress response pathways. This shift underscores the significant impact of biotic stress on the metabolic network within the pepper, particularly in how it alters the balance between growth and defense. Therefore, based on their discoloration or unusual shape, biotic-stressed Subicho red peppers were hypothesized to have undergone a change in metabolite flow due to prolonged stress. Such insights are crucial for effectively developing strategies to manage both the quality and disease resistance of the fruit [31].

In this study, metabolite components, including the key amino acids, were also observed to have changed. Increased levels of amino acids, such as threonine, glycine, serine, alanine, asparagine, aspartic acid, γ-aminobutyric acid, tyrosine, phenylalanine, and tryptophan, as well as increased levels of fatty acids, acetic acid, citric acid, and succinic acid, indicated the widespread activation of various metabolic pathways during stress. Among the amino acids, serine, glycine, and threonine increased protein synthesis and turnover and enhanced nitrogen assimilation via aspartic acid and asparagine. Alanine was also predicted to be present in large amounts because it often accumulates in nitrogen metabolism and functions as a signaling molecule. This is thought to be the result of increased protein synthesis and other nitrogen-demanding processes during stress to maintain plant homeostasis [32]. The levels of aromatic amino acids, such as tyrosine, phenylalanine, and tryptophan were also higher in the biotic-stressed Subicho red peppers compared to the healthy Subicho red peppers. This phenomenon was possibly the result of the increased production of secondary metabolites via the shikimate pathway, which plays an important role in plant defense and stress signaling [33]. These amino acids not only contribute to protein synthesis but are also precursors for various secondary metabolites, many of which are involved in the defense of the plant against pathogens. The observed increase in the levels of pathogens suggests an intensified metabolic response, aimed at bolstering the defenses of the pepper against biotic stress. As a representative of stress-producing substances, the levels of γ-aminobutyric acid were also higher in the biotic-stressed Subicho red peppers, suggesting that this acid plays an important role in stress response mechanisms, signal transduction, and cellular pH regulation [34]. Compared to the healthy Subicho red peppers, the biotic-stressed Subicho red peppers had higher levels of acetic acid, citric acid, and succinic acid, which are associated with the central metabolism and the tricarboxylic acid cycle. According to the results of Choi, et al. [35], increased levels of citric and succinic acid reveal an up-regulation of the Krebs cycle, thereby meeting the increased demand for energy and biosynthetic precursors induced by stress. This up-regulation of the TCA cycle and related pathways likely reflects the increased need of the pepper for ATP and the metabolic intermediates that are critical for sustaining the enhanced biosynthetic and defensive activities under biotic stress conditions. Moreover, in a study by Vo, et al. [36], plantlets infected with the fungus that causes sheath blight in rice induced apoptosis from the onset of infection. They also induced glycolysis and TCA cycle intermediates but had reduced levels of sugar metabolites. These results were probably due to the increased energy production in the infected tissues and were similar to those of the present study. Moreover, acetic acid probably plays an important role as a precursor to acetyl-CoA, which is indispensable for energy production and the biosynthesis of essential metabolic intermediates, thereby replenishing the demand for biosynthetic precursors [37].

In addition to the changes in the amounts of the major metabolites, changes in the fatty acid composition of the biotic-stressed Subicho red peppers were observed. These changes suggested alterations to the membrane lipids, which are crucial for maintaining cell integrity under the conditions of pathogen attack and stress [38]. Moreover, they are essential for enhancing plant resistance, optimizing defense mechanisms, and ensuring efficient energy management under adverse conditions. Therefore, in contrast to the metabolic changes typically observed in harvested red peppers, those subjected to biotic stresses exhibited numerous complex alterations. These findings highlight the extensive metabolic reprogramming that occurs in response to biotic stress, triggering a coordinated defense response involving multiple metabolic pathways. These insights into the mechanisms by which peppers are able to withstand pathogen attack are pivotal for breeding programs aimed at enhancing disease resistance and can ultimately improve the crop productivity and utility to increase their value as food.

## 3. Materials and Methods

### 3.1. Plant Source and Sample Preparation

In this study, red peppers (*Capsicum annuum* var. *annuum*) were cultivated under standard farming practices at the Yeongyang Pepper Research Institute (Yeongyang, Republic of Korea) and harvested on 10 August 2023. Subicho, a traditional Korean landrace variety known for its balanced spicy and sweet flavor profile, has been cultivated in the Yeongyang region since before 1950 [39]. This variety is prized for its high milling rate and superior quality. The peppers were sorted into two distinct groups for analysis: healthy Subicho peppers and biotic-stressed Subicho peppers. The healthy Subicho peppers were cultivated in an open field under controlled conditions at the Yeongyang Pepper Research Institute. The soil was slightly acidic (pH 6.5) and was composed of loam or clay loam with good drainage and a high water retention capacity. During cultivation, temperatures ranged from 25 to 28 °C during the day and 18 to 22 °C at night, with the lowest recorded temperature not falling below 13 °C. The daytime soil temperature was between 18 °C and 28 °C. Regular watering ensured optimal moisture levels, as the plants are sensitive to both drought and flooding. In contrast, the biotic-stressed Subicho peppers were cultivated in the same open field but were naturally exposed to biotic stress. Anthracnose, a common fungal disease in pepper crops, was the primary biotic stress observed. The Yeongyang Pepper Research Institute selected the samples based on visible symptoms, including surface blemishes, discoloration, deformities, and the characteristic signs of anthracnose, such as water-soaked lesions and pink fungal growth on the fruit [40,41]. These peppers were collected specifically to study the metabolic differences induced by natural biotic stress factors. To ensure a comprehensive analysis, ten biological replicates were prepared for each group. After harvesting, the placenta and seeds were manually removed from the pepper samples. The pericarps were immediately frozen in liquid nitrogen and stored at −80 °C until further processing. For the metabolomic analysis, the frozen pericarps were ground using an analytical grinding mill (Model A 11, IKA Works Inc., Staufen im Breisgau, Germany), transferred to Eppendorf tubes, and stored at −80 °C for 24 h. The samples were subsequently freeze-dried for 48 h.

### 3.2. ^1^H NMR Spectroscopic Analysis of the Red Pepper Extracts

For the ^1^H NMR analysis, metabolites were extracted from the samples obtained from the red pepper pericarps according to the methods of Kim, et al. [42] and Seong, et al. [43]. The deuterated methanol-d_4_ (CD_3_OD, 99.8 atom% D), potassium dihydrogen phosphate (KH_2_PO_4_), sodium deuterium oxide (NaOD), and deuterium oxide (D_2_O, D 99.9 atom%) were supplied by Sigma-Aldrich (St. Louis, MO, USA). A buffer was prepared by adding KH_2_PO_4_ (1.232 g) to D_2_O (37.5 mL), and the pH was adjusted to 6 using NaOD, followed by the addition of CD_3_OD (62.5 mL). Each freeze-dried sample (20 mg) was dissolved in an Eppendorf tube containing the prepared buffer (1 mL). The resulting mixture was sonicated for 20 min at 25 °C to extract the metabolites from the red pepper and centrifuged at 19,083× *g* for 20 min at 4 °C. The supernatant from each red pepper extract (550 μL) was subsequently transferred to 5 mm NMR tubes. A quality control (QC) sample was prepared by combining equal volumes of all the red pepper extracts for the NMR analysis [44]. This QC sample was used to monitor instrument performance and further consider the ^1^H–^1^H total correlation spectroscopy (TOCSY) and heteronuclear single-quantum correlation (HSQC) spectra. The CD_3_OD was used as a field frequency lock in the supernatant, and the signal of the methyl group (methanol-d_4_) was used as a chemical shift reference (^1^H, δ 3.324). The ^1^H NMR spectra were acquired on a Bruker Avance 700 spectrometer (Bruker Biospin, Rheinstetten, Germany), operating at a proton frequency of 700 MHz and a temperature of 298 K. The spectrometer was equipped with a cryogenic triple-resonance probe and a Bruker automatic injector. One-dimensional (1D) ^1^H-nuclear Overhauser effect spectroscopy (NOESY) was performed using a pulse sequence from the Bruker library (noesygppr1d). The two-dimensional (2D) TOCSY and ^1^H–^13^C HSQC spectra were acquired using dipsi2esgpph and hsqcetgpsisp2 pulse sequences, respectively, from the Bruker library.

### 3.3. NMR Data Processing and Multivariate Statistical Analysis

All ¹H NMR spectra were manually calibrated using methanol-d_4_ (^1^H, δ 3.324) and adjusted to correct phase shifts and baseline irregularities using Topspin software (version 4.3.0, Bruker Biospin, Rheinstetten, Germany). Subsequently, the calibrated and corrected spectra were imported into MATLAB (R2014a; The Mathworks Inc., Natick, MA, USA) and further aligned using the icoshift and correlation-optimized warping methods [45]. The spectra were normalized using total area and quotient probabilistic methods to avoid dilution effects. The multivariate statistical analysis (MVA) of full resolution ^1^H NMR spectra was performed without spectrum bucketing or binning and excluded the unnecessary regions from δ 0 to 0.5, 3.31 to 3.33, and 9.6 to 10 ppm. The MVA of the integral datasets of assigned metabolites was further performed and visualized using orthogonal projection to latent structure discriminant analysis (OPLS-DA) score plots. The resulting datasets were imported into SIMCA (version 18.0; Sartorius Stedim Biotech, Umeå, Sweden) for MVA using a mean-centered scaling method [46]. OPLS-DA eliminates systematic variations from the input data matrix X (representing compounds or metabolites) that are unrelated to the response matrix Y (discriminant classes). This method was applied as a supervised pattern recognition technique to extract the relevant information from the discriminant compounds within the dataset. Hotelling’s *T*-squared distribution (95%) was calculated in SIMCA to identify strong outliers within each sample to ensure that all the available data were within the 95% confidence interval. The OPLS-DA models were constructed using seven-fold cross-validation and a permutation test with 200 iterations. To improve the interpretation of the results and pinpoint metabolites that contributed to the difference between the two groups, OPLS loading or coefficient plots were created with color-coded correlation coefficients for each data point, utilizing MATLAB scripts developed at Imperial College London. The models’ quality was assessed using the R^2^X, R^2^Y, and Q^2^ values, where R^2^X is the proportion of variance in the data explained by the models, indicating the goodness of fit, R^2^Y is the extent to which the model explains the variance in the dependent variable, and Q^2^ is the proportion of the variance in the data predicted by the model. A relative quantification of the metabolites was performed using the integral area of each corresponding peak of the metabolite in the ^1^H NMR spectra. Table S1 presents the signal ranges used, and those that overlapped with other metabolites were excluded from the analysis.

### 3.4. Statistical Analysis

All the obtained results were expressed as mean ± SDs using at least ten independent measurements. The paired Student’s *t*-test was used to confirm the significance of the metabolite differences observed in the OPLS coefficient or loading plots for pairwise comparison. The relative quantification of the metabolites in the ^1^H NMR spectrum was calculated with the integral area of each peak corresponding to a metabolite (* *p* < 0.05, ** *p* < 0.01, and *** *p* < 0.001).

## 4. Conclusions

NMR spectroscopy was utilized to investigate the metabolomic profiles of fully mature healthy and biotic-stressed Subicho red peppers, a traditional Korean landrace variety. The results revealed significant metabolic differences between healthy and biotic-stressed Subicho red peppers and highlighted the biochemical impacts of biotic stress on this heritage crop. The biotic-stressed Subicho red peppers, the unfavorable visual appearance and flavor of which lowers their market value, exhibited lower sucrose levels in their pericarps, suggesting a shift from sugar storage to defense mechanisms. In contrast, elevated levels of stress-associated amino acids such as valine, leucine, and isoleucine, along with TCA cycle metabolites that include acetic, citric, and succinic acids, as well as nitrogen-related compounds including alanine, asparagine, and aspartic acid, indicated a broad activation of the metabolic pathways essential for maintaining growth and defense under stress conditions. These findings provide crucial insights into the metabolic adaptations of Subicho in response to biotic stress and could potentially be incorporated into breeding and post-harvest strategies aimed at improving stress resistance and quality. Despite the value of the data produced in this study, it is important to acknowledge the limitations, such as the relatively small number of metabolites analyzed and the limited size of the dataset. Additionally, the results are specific to Subicho and address general biological stress responses rather than isolating individual stressors, thereby limiting the broader applicability of the results across other species or types of stress. Future studies aim to focus on expanding the metabolite profile and to explore specific biotic stressors to deepen our understanding and enhance the generalization of the findings.

## Data Availability

The original contributions presented in the study are included in the article/Supplementary Materials. Further inquiries can be directed to the corresponding author.

## Supplementary Materials

The following supporting information can be downloaded at https://www.mdpi.com/article/10.3390/ijms25189903/s1.
