# Supplementary material for: NMR-Based Metabolomic Analysis of Biotic Stress Responses in the Traditional Korean Landrace Red Pepper (Capsicum annuum var. annuum, cv. Subicho)
Source: Int J Mol Sci. 2024 Sep 13;25(18):9903. doi: 10.3390/ijms25189903 (PMC11432352; doi:10.3390/ijms25189903)
Supplement: Supplementary file 1 [file ijms-25-09903-s001.zip › ijms-3179693-supplementary.pdf]

Supplementary Materials

Table S1. Metabolites identified in the red pepper specimens.

No.	Abbreviation	Metabolite	δ¹H (ppm)	δ¹³C (ppm)	Multiplicity
1	α-Glc	α-Glucose	5.18	95.16	d
			3.82	55.44	d
			3.79	84.14	m(OVLP)
2	β-Glc	β-Glucose	4.57	99.23	d
			3.70	76.03	dd
3	Suc	Sucrose	5.41	95.05	d
			4.16	79.88	d
			4.03	76.07	s(OVLP)
4	Fru	Fructose	4.08	66.15	dd(OVLP)
			3.74	66.45	m(OVLP)
5	Tre	Trehalose	3.88	63.95	m
6	Thr	Threonine	4.21	68.76	d(OVLP)
			3.48	63.44	d(OVLP)
			1.34	22.51	d(OVLP)
7	Ser	Serine	3.75	59.47	dd(OVLP)
			3.91	63.22	m(OVLP)
8	Asn	Asparagine	2.96	37.23	dd
9	Gln	Glutamine	2.47	33.88	m
			2.14	29.23	m
			3.70	57.10	t(OVLP)
10	Ala	Alanine	1.50	18.97	d
			3.70	53.40	q(OVLP)
11	Gly	Glycine	3.49	44.15	s
12	Pro	Proline	4.12	61.24	dd
			2.34	31.91	m(OVLP)
13	Val	Valine	1.04	20.86	d
14	Leu	Leucine	3.70	57.10	m(OVLP)
			1.76	23.49	m(OVLP)

			0.98	23.64	d(OVLP)
15	Ile	Isoleucine	1.02	17.35	d
			1.98	34.66	m
16	Asp	Aspartic acid	2.72	39.26	dd
			2.81	39.25	dd(OVLP)
17	GABA	γ-Aminobutyric acid	3.01	42.21	t
			2.32	37.44	t
			1.91	26.46	q(OVLP)
18	Arg	Arginine	3.24	43.41	t
			1.92	30.91	m(OVLP)
19	Phe	Phenylalanine	7.34	132.09	m
			7.33	130.26	d(OVLP)
			7.39	131.76	m
20	Trp	Tryptophan	7.72	121.06	d
			7.45	114.29	d
21	Tyr	Tyrosine	7.18	133.40	d(OVLP)
22	AcOH	Acetic acid	1.91	26.43	s(OVLP)
23	FoA	Formic acid	8.49	172.41	s
24	MA	Malic acid	4.29	72.48	dd(OVLP)
			2.74	44.69	dd(OVLP)
25	SA	Succinic acid	2.42	36.76	s
26	GA	Glutaric acid	2.32	37.40	t
			1.91	26.44	tt(OVLP)
27	CA	Citric acid	2.64	47.41	d(OVLP)
			2.73	47.41	d(OVLP)
28	QA	Quinic acid	4.12	73.23	ddd(OVLP)
			2.48	41.37	dd(OVLP)
29	HBA	Hydrobenzoic acid	7.69	134.54	d
			6.95	117.80	d(OVLP)
30	Cin	Cinnamate	7.61	130.47	m
31	Chol	Choline	3.22	56.55	s
			4.06	58.47	m
32	TG	Trigonelline	9.37	145.46	s

			9.13	145.47	d
			9.04	150.31	d
			8.23	130.44	m(OVLP)
33	MEA	Ethanolamine	3.06	46.03	t
			3.75	59.59	t
34	Ino	Inosine	8.32	142.53	s
35	3-IS	3-Indoxylsulfate	7.38	118.96	t
36	Urd	Uridine	5.89	92.12	d
			7.86	144.56	d
37	Ac-Tyr	Acetyl-tyrosine	7.05	118.96	m
38	$NADP^+$	Nicotinamide adenine dinucleotide phosphate	5.98	102.83	dd
39	ST	Sterols	0.80	18.63	d
40	GL	Glycerol	3.77	74.97	m(OVLP)
			3.65	65.48	dd
41	FAs	Fatty acids	1.27	31.78	m
42	BA	Butyric acid	0.85	14.39	t(OVLP)
			1.49	18.98	m(OVLP)
			2.11	42.31	t(OVLP)

Multiplicity abbreviations: s = singlet, d = doublet, t = triplet, q = quarter, m = multiplet, OVLP = overlapping signals.

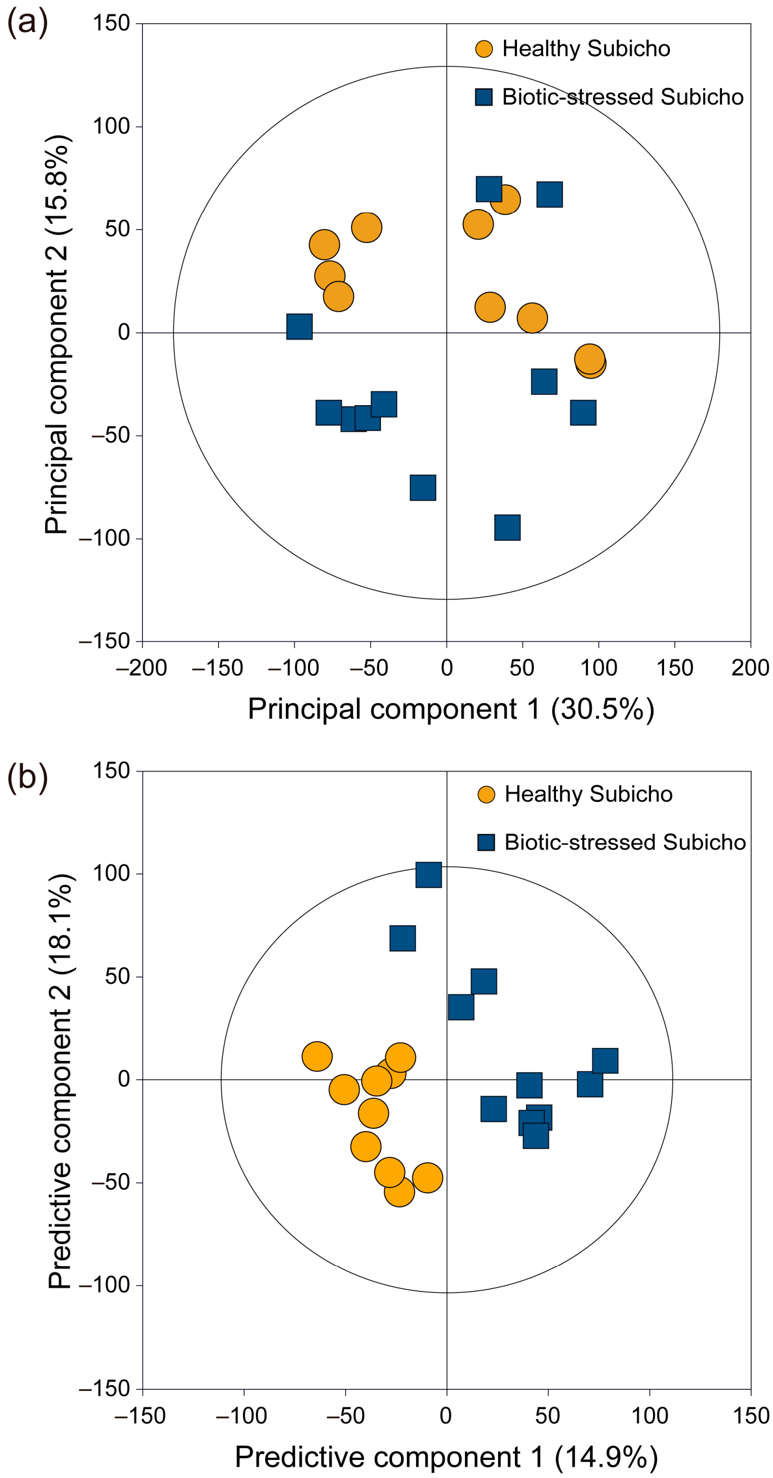

Figure S1. (a) PCA score plot and (b) PLS-DA score plot between healthy Subicho and biotic-stressed Subicho red peppers derived from the ^{1}H NMR spectra (700 MHz).

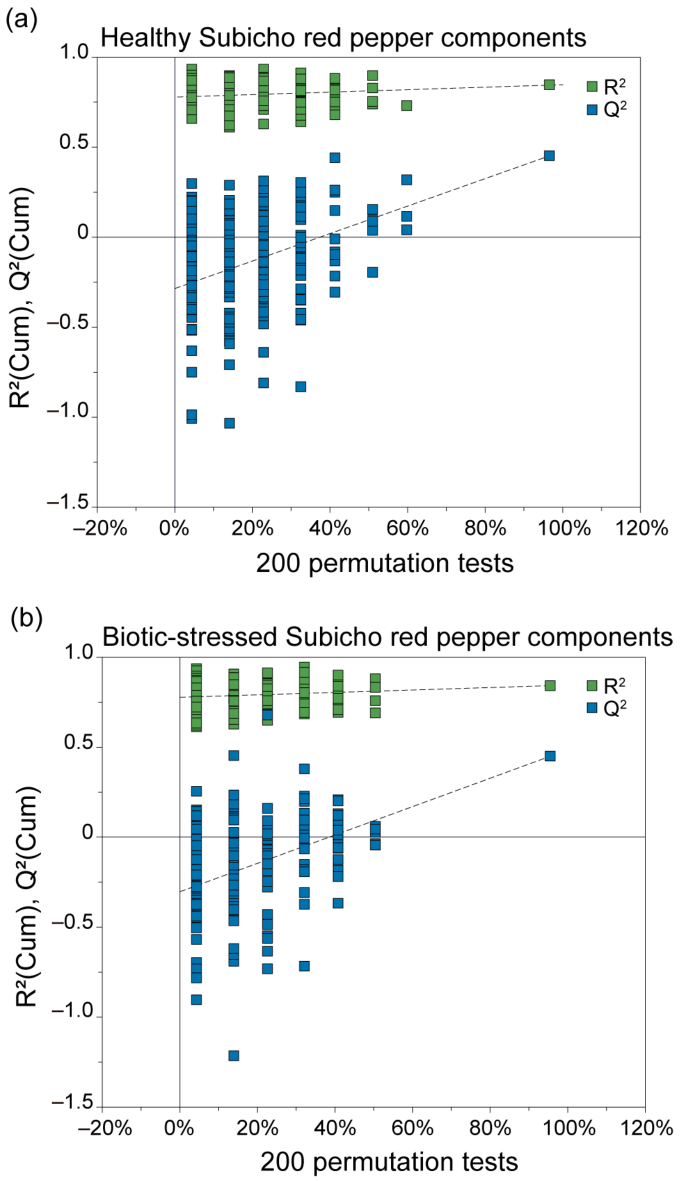

Figure S2. Permutation test confirming the discrimination between healthy Subicho (a) and biotic-stressed Subicho (b) red pepper using the OPLS-DA model shown in Figure 3.
